# Increasing challenges of general practitioner-oncologist interaction in end-of-life communication: a qualitative study

**DOI:** 10.1186/s12904-025-01690-w

**Published:** 2025-02-20

**Authors:** Matthias Villalobos, Anastasia Korezelidou, Laura Unsöld, Nicole Deis, Michael Thomas, Anja Siegle

**Affiliations:** 1 Department of Thoracic Oncology, University Hospital Heidelberg and Translational Lung Research Center Heidelberg (TLRC-H), Member of the German Center for Lung Research (DZL), Heidelberg, Germany; 2 Faculty of Business and Health, Department of Health Sciences and Management, Baden- Wuerttemberg Cooperative State University Stuttgart (DHBW), Stuttgart, Germany

**Keywords:** End-of-life communication, Interdisciplinary communication, Cancer care coordination, Primary care, Palliative care

## Abstract

**Background:**

The timely initiation of end-of-life (EOL) conversations is considerered best practice for patients with advanced cancer and therefore recommended in oncological guidelines. General practitioners (GPs) play a key role in the coordination of EOL-care and often claim that they have also the responsibility to initiate the necessary conversations. Nonetheless, the content of these conversations is rarely a subject of communication between GPs and oncology specialists but crucial for timely integration of palliative care. The aim of this study is to explore the GPs‘ perspectives on initiation and conduction of EOL-conversations in patients with metastatic lung cancer and how they perceive the interaction with the oncologists of a cancer center.

**Methods:**

Qualitative design with in-depth interviews with GPs that refer patients with metastatic lung cancer to a cancer center; thematic analysis following Braun and Clarke.

**Results:**

We identified three main themes: timing and conduction of EOL-conversations, factors influencing EOL-conversations, and modes of GP-oncologist interaction. All themes showed important and increasing challenges in regard to communication strategies or procedures within the cancer center and between general practitioners and oncologists. Aside from the elucidated challenges of EOL-communication, new problems arise from the difficulties in keeping pace with advances in oncology and the increasing prognostic uncertainty. Additionally, the lack of standardized communication in EOL-topics between GPs and oncologists is underlined. Options in the mode of interaction include written information in reports or digital platforms and direct phone calls.

**Conclusions:**

Because of the growing complexity in modern oncology, strategies for improvement in GP-oncologist interaction have to consider information about therapeutic advances and prognosis of patients. The increasing prognostic uncertainty hazards the adequate provision and conduction of EOL-conversations and thus, the timely integration of palliative care. As a consequence, a trustful personal interaction that includes direct contact via phone calls between GPs and oncologists should be encouraged.

**Supplementary Information:**

The online version contains supplementary material available at 10.1186/s12904-025-01690-w.

## Introduction

The timely initiation of end-of-life (EOL) conversations is considerered best practice for patients with life-limiting disease and therefore recommended in medical guidelines [1, 2]. These conversations between health care professionals, patients and their relatives concerning their values and wishes should be part of the care planning and include topics like prognosis, treatment preferences, preferred place of death, communication with relatives, symptom management, advance directives, and psycho-social and existential issues. Early conversations improve satisfaction in patient-clinician communication, quality of life, timely integration of palliative care and consequently reduce futile aggressive EOL-care [3]. In this regard, the facilitation of prognostic awareness is considered fundamental for goal-concordant care in EOL-communication. Many factors have been identified influencing whether EOL-conversations are initiated: time constraints, concern about patients‘ reaction, cultural issues, individual physicians‘ experiences and prognostic uncertainty, among others [4, 5]. General practitioners (GPs) play a key role in the coordination of EOL-care and often claim that they have also the responsibility to initiate the necessary conversations [5]. Primary care serves best to this purpose because of its strengths in continuous and comprehensive care. A recent study corroborates this assumption: cancer patients mentioned most commonly their primary care provider as the preferred contact for these conversations and wished the initiation early in the disease [6]. Nonetheless, the growing complexity in the health care system and particularly in the care of patients with cancer, pose a continuous threat to good coordination and communication. Lung cancer is associated world-wide with the highest cancer-related mortality and poses specific challenges to EOL-conversations: a mostly elderly and frail population that in decision-making is exposed to the continuous balancing of hope in the rapidly increasing new treatment options with the timely integration of palliative care [7, 8]. In this setting, the relationship between primary care providers and oncology specialists is of essential importance for the coordination of care during the whole cancer disease trajectory but also poorly understood. Although oncologists recognize the GPs’ central role in holistic care, they may also see them as “risk factors” in their relationship with patients if they challenge their treatment decisions [9, 10]. Prior research shows the need for improved definition and coordination of roles and responsibilities [11]. When asked about perceived needs of care coordination, patients indicate that communication plays a key role. On the one hand, good communication between patients and health care providers about treatment goals leads to increased satisfaction with care. On the other hand, gaps in communication between providers lead to discontent, poor care confidence and a feeling of abandonment at the worst [12]. Content of EOL-conversations are rarely a subject of communication between GPs and oncology specialists. Coordination on who discusses what and when is mostly neglected or even completely absent [13].

The aim of this study was to explore GPs‘ perspectives on initiation and conduction of EOL-conversations in patients with metastatic lung cancer and how they perceive the interaction with the physicians of the cancer center.

## Methods

We used a qualitative design with in-depth interviews with general practitioners. Based on literature a semi-structured interview guide was developed (additional file 1) and pre-tested with one physician. Two researchers (AK and AS) who were unknown to the participants conducted the interviews until recurring themes indicated saturation of data from August 2020 to October 2021 by telephone because of contact restrictions during the Covid19-pandemics. The longer recruitment period was also due to the pandemics. The interviews lasted between 25 and 43 min (average 32 min), were audio-recorded and transcribed verbatim using F4 software [14].

Data analysis was performed following thematic content analysis by Braun and Clarke, taking into account a realist approach [15]. Pragmatism was chosen as epistemological approach. In pragmatism the emphasis lies on actionable knowledge [16]. Two researchers (AS and AK) followed the six steps independently from each other. Diverging codes were discussed in the team with the principal investigator (MV) until full agreement and at an international qualitative research workshop for feedback on the results. For data management and analysis we used MAXQDA 12 software (version 12.3.9). The reporting follows COREQ criteria [17]. This study was conducted at the Department of Thoracic Oncology of Heidelberg University (Germany’s largest lung cancer center and founding member of the National Center for Tumor Diseases NCT). Ethical approval was given by the Ethics Committee of the Faculty of Medicine of Heidelberg University (S-478/2020). Written informed consent was obtained from all participants and their pseudonymization and confidentiality was ensured throughout the study.

## Results

We conducted interviews with 10 general practitioners from urban and rural settings with characteristics of participants shown in Table 1. We identified 3 main themes: timing and conduction of EOL-conversations, factors influencing EOL-conversations, and modes of GP-oncologist interaction. All themes showed important challenges in regard to communication strategies or procedures within the cancer center and between general practitioners and oncologists (Table 2).

**Table 1:** Characteristics of participating general practitioners (*n* = 10)

Characteristics	Number	Range
Sex -female -male	6 4	
Age (mean in years)	54	44–66
Professional experience (mean in years)	17	6–33
Patients treated per quarter (mean)	2685	1200–3759

**Table 2:** Main themes

Themes	GP perspective	Challenges in regard to communication in and with the cancer center
Timing and conduction of EOL-conversations	Should be offered early in the disease	Most GPs see the responsibility to start the conversation in the hands of the center’s physicians
	Importance of honest information	GPs often lack information about prognosis and content of diagnosis disclosure in the center
	Individual approach depending on patient’s preparedness	Varying communication partners (physicians and other health care professionals) in the center with different attitudes
	Concern of patient’s reactions/fear to destroy hope	GPs often do not interfere with center’s treatment decisions
	Patient’s process of assimilation of information and coping with the disease	GPs are often not involved in the process during cancer treatment
	Important role in EOL-care (support/coordination)	Abrupt shift to GP after end of cancer treatment
Influencing factors of EOL-conversations	Lack of specific medical knowledge (because of rapid advances in oncology)	Insufficient medical information given to the GP by the center
	Growing prognostic uncertainty (because of rapid advances in oncology)	Vulnerable patient-physician communication in the center: risk of overly optimistic communication
	Steady communication partner, longer relationship with patient	Varying communication partners in the center
	Sometimes different opinion between GP and oncologist about treatment goals	Difficulties reaching center’s doctors, patient-empowerment as an option to get involved
Modes of GP-oncologist interaction on EOL-topics	Medical reports	Useful update about treatment options, lack of information about EOL-topics
	Collaborative use of digital platforms	Not generally implemented
	Phone calls	Difficulties reaching center’s doctors
	Patient as intermediary	Complicated role for patient

1. Timing and conduction of EOL-conversations:

In general, GPs emphasize that these conversations should be offered early in the disease and that honest information is important. One GP uses very open and direct ways of communication. *„I’m an advocate of the truth. I don’t encourage unrealistic hopes*,* but tell them [the patients] that they have an incurable disease.“ [GP 3*,*5] „ It’s my attitude that the patient has a right to know the truth. Even if he doesn’t want to hear it.“ [GP 3*,*55]*.

More GPs choose a rather cautious approach depending on the patient’s preparedness and try not to overwhelm with information.

*„You don’t have to say everything always. There are patients who want to hear little or nothing.“ [GP 1*,*13]*.

Another GP explains: *„It’s step by step. A process that evolves. […] I know patients that shut down and then I don’t push them to hear it.“ [GP 11*,*20]*.

In general, GPs express difficulties of EOL-communication, most importantly the concern for emotional reactions and the fear to destroy hope.

*„Actually*,* we also find it difficult to say: in this case we would stop the cancer treatment.“ [GP 10*,*35]*.

The responsibility for the initiation of EOL-conversations and the allocation of specific topics seems not to be clearly defined. Several GPs state that it is the oncologist’s responsibility to address prognosis and limitations of treatment/advance directives.

*„I see it clearly with the specialists. To say*,* we can still do something or not.“ [GP 4*,*52]*.

Other GPs position themselves in a stronger role. This includes not only the initiation of these conversations but also the role in supporting and reinforcing communication during the whole course of disease, incorporating and coordinating where needed specialized psycho-social support and palliative care teams. They emphasize their abilities in using more patient-centered language, their long-standing trustful relationship with the patient and their better knowledge about his social environment.

*„Everything that was discussed in the hospital*,* they want it discussed again with the GP. […] I see myself as the one closer to the patient. Who perhaps enjoys more trust.“ [GP 6*,*39]*.

Still, challenges arise depending on how these topics were communicated with the patient in the cancer center. GPs describe situations of conflict with perceptions of overly optimistic communication or the suspected withholding of information in the cancer center.

*„It always depends on how the first conversation [in the cancer center] went. Did they fuel too much hope…“ [GP 4*,*55]*.

2) Factors influencing EOL-conversations:

GPs clearly describe that even when feeling the responsibility to start EOL-conversations, the lack of specific medical knowledge may be a hindering factor.

*„These [cancer] therapy regimes*,* […] what is still possible*,* and what is reasonable. In this*,* as GPs*,* we are not that fit anymore nowadays. It’s so complex*,* the third antibody or the third checkpoint-inhibitor for lung cancer*,* I don’t know it.“ [GP 10*,*33]*.

Because of the knowledge gap it is difficult for GPs to determine the risk-benefit ratio or to estimate the end of a cancer treatment. The rapid changes in oncological treatment options may even lead to avoidance of talking about prognosis and EOL at all and the risk of supporting unrealistic expectations.

*„In most conversations I leave it [the prognosis] always open in the beginning. And I say that nobody knows how long it will take. Because we have new findings every day*,* new drugs are being approved.“ [GP 8*,*49]*.

On the one hand, GPs describe a relation of trust with the oncologists and a strategy of not interfering with the center’s care.

*„I rely on the expertise of the colleagues in the hospital of course. Normally I follow the hospital’s recommendations.“ [GP 3*,*33]*.

On the other hand, some situations seem problematic when opinions about treatment goals differ. Some GPs choose patient-empowerment as a strategy.

*„And there to be the advocate*,* that someone can live as he wants. If he still wants to go on a trip I say: discuss with your oncologist if a treatment break makes a difference or not. […] I encourage to ask questions.“ [GP 10*,*37]*.

Another strategy is to approach the oncologist directly to express their view. But this seems to happen merely when general practitioner and oncologist are acquainted with each other. Otherwise, they try to navigate the dilemma between conflicting opinions or attitudes.

*„When the oncologist has a different perception and we see it more pessimistically and then try to accompany with palliative care… that is often difficult.“ [GP 4*,*31]*.

Often GPs are left to wait until the cancer center takes the decision to stop the oncological treatment.

*„It isn’t over until the hospital says: we have no treatment options left.“ [GP 8*,*49]*.

As cancer treatment may expand over multiple therapy lines it is uncertain when options will be exhausted, also impairing the initiation of EOL-conversations. GPs describe the lack of transparency about what was discussed exactly with the patient at the cancer center.

*„Let’s say*,* I assume that the patient has been told the basics*,* you have this and that*,* the prognosis is this. I assume it*,* but of course I don’t know it for certain.“ [GP 11*,*40]*.

The patient may function as an intermediary for the missing information. But GPs say that sometimes it is difficult to rely on patients‘ statements because of the information’s complex and sensitive nature.

*„I mean*,* sometimes you get strange answers from the patients*,* that*,* we all know*,* are completely different from what was said. So*,* they hear what they want to hear.“ [GP 8*,*57]*.

GPs emphasize that the disease understanding and coping process may take some time and that they play an important role in repeating and explaining the center’s information. They often have a much longer and continuous relationship with the patients and their families, while communication partners in the hospital vary constantly. All these aspects are underlined as of special importance in the delivering of bad news and EOL-communication.

*„Patients understand*,* they listen*,* but not all information reaches them. And you have to tell some things repeatedly. Again and again. That is*,* I think*,* completely normal. They are often overwhelmed*,* aren’t they? With all this information they get.“ [GP 6*,*23]*.

Nonetheless, GPs often lose some or complete contact with cancer patients during the active treatment phase because of the close connection with the center with regular visits for therapy and follow-up. When cancer treatment comes to an end, care coordination is expected to shift back to the GP but this may happen abruptly. This again may hinder adequate EOL-care.

*„The more terminal it [the disease] becomes at the end*,* the more it will be me who takes over the care*,* right?“ [GP 5*,*30]*.

3) Modes of GP-oncologist interaction:

The official way of receiving medical information from the hospital is the written report that is handed to the patient at discharge after hospital treatment or at a follow-up visit in the outpatient department. The oncological information is considered very useful by the GPs even for professional medical update (e.g. new cancer treatments). This differs considerably from the information about the content of prognosis communication and EOL-conversations.

*„This is always a little bit difficult. To talk to the patient about prognosis when I don’t have all the information or I don’t know what is still planned generally.“ [GP 4*,*31]*.

*„We can’t estimate the prognosis that well. This we have to leave to the center. But it would be good if we then would receive some information. Sometimes we find it out when talking to the patient.“ [GP 8*,*21]*.

GPs express different opinions when deliberating on which mode of interaction with oncologists about EOL-communication could be the most appropriate. Some state that written information is enough, including digital options.

*„Well*,* a phone call is not necessary for this. But a short notice „advance directive was made“ or „palliative situation was discussed“*,* that would be good.“ [GP 1*,*41]*.

*„On the long run I would say that everything should be placed on a digital platform. […] It would be ideal and handy if the center would be logged in*,* too.“ [GP 10*,*47]*.

Nonetheless, it is of concern to GPs that the information and communication load is already overwhelming even without these additional topics.

For this reason some GPs say that a phone call would be the best solution. Still, challenges arise when considering the time limitations in the GPs‘ and hospital clinicians‘ workday, sometimes also differing in working hours and consequently the difficulties in reaching each other.

*„We would wish that we could talk to the colleague also personally. Because it’s a very important phase of course. We tried everything at the beginning*,* yes. And now we see that the tumor grows and new metastases appear. And the patient gets sicker after the therapy. Then we should discuss: how far do we want to go? […] Then it would be good if the colleague called us and told us his perception. So that we have a better basis for our conversation with the patient.“ [GP 4*,*45]*.

Prognosis and EOL-topics are not only difficult to discuss but also the documentation and information of GPs are challenging.

*„Yes*,* exactly about this prognosis-factor we should be informed. But*,* I don’t have a good idea at the moment how to do it.“ [Niko 8*,*57]*.

Still, the importance to achieve an optimal information flow about this topic is emphasized. This may improve the relationship between GP and oncologist, the communication with patients and ultimately the patients‘ care.

*„If both of us deliver our conversations in unison*,* it’s also easier. […] It would certainly be good for the patient if we both say the same thing*,* right?“ [GP 5*,*32]*.

*„And then it’s also good to grab the phone and say [to the oncologist]: let’s talk briefly and honestly. […] This is sometimes also this challenging borderline area*,* so that we should relate to each other trustfully*,* that we both mean it well.“ [GP 10*,*57]*.

## Discussion

This study explores the complex topic of EOL-communication in lung cancer patients whose care is typically shared between general practitioners and oncology specialists. It exposes from the GP perspective various challenges and conflicts regarding the interaction between physicians that jeopardize the timely integration of palliative care.

GPs express the difficulties of EOL-communication between patients and health care providers as described by previous research [18]. The fear to destroy hope and to deteriorate the patients‘ emotional condition are the most important challenges mentioned. In contrast, evidence suggests that the correct conduction of EOL-conversations does not harm and can be beneficial for the GP-patient relationship [19, 20]. Nonetheless, the GPs in our study express a strong commitment and feeling of responsibility to offer these conversations and emphasize that communication should be tailored to the patients‘ preparedness.

Our study reveals new challenges due to the increasing prognostic uncertainty and the struggle to keep up with the pace of therapeutic developments in oncology. Prior research shows that oncology and non-oncology doctors‘ estimates of prognosis of cancer patients are mostly inaccurate and that of non-oncologists (including GPs) is considerably over-pessimistic [21, 22]. In the worst case this may lead to undertreatment and affect survival. As a consequence, several GPs in our study shift the responsibility to provide this information completely to the oncologists. Nonetheless, the GPs are certainly involved in the communication with the cancer patients and engaged in reformulating and explaining the medical information received in the center. Uncertainty about what was said exactly by the oncologists appears as an additional stressor as this information is not documented or communicated most of the time. Several studies underline not only the GPs‘ need to receive adequate and timely information from the hospital team but also the often ineffective communication that leads to disruptive care coordination [23–25]. As in our study, specifically EOL-topics and the content of respective conversations are rarely a subject of interdisciplinary interaction and the patient often finds himself in the role of intermediary [13]. This role is complicated as our study participants also describe the fact that illness understanding and coping may vary significantly between individual patients. Information overload combined with the emotional reaction to cancer diagnosis and distress related to having an advanced disease may lead to the inability to assimilate the news or even the rejection to further talk about the topic. GPs emphasize the importance of their long-standing relationship with the patients compared to the cancer center where communication partners may vary. This is an important aspect because this discontinuity affects both the patients and the GPs. Also from the ethical perspective prognosis disclosure needs an individualized and cautious approach that can only be determined by the relationship fostered over time with patients [26]. Complicating, as in our study, GPs express that during cancer treatment they often lose contact with patients and involvement in care declines [11, 24]. Often GPs wait for the oncologist to finish cancer treatment to be able to start EOL-conversations. This may happen abruptly hazarding the integration of palliative care. In metastatic lung cancer, the positive evidence of early integration of palliative care led to a respective recommendation of the American Society of Clinical Oncology in 2012 and found afterwards its way into the oncological guidelines [27, 28]. However, in the last decade the therapeutic algorithms in lung cancer treatment have changed dramatically because of the continuous approval of new drugs, mostly targeted therapies and checkpoint-inhibitors [7]. This has led to increasing prognostic uncertainty and new challenges in patient-physician communication [29]. For certain, to offer EOL-conversations has become recommended standard of care in palliative oncology. Still, the implementation remains challenging as new treatment options always risk to push EOL-communication and the integration of palliative care aside as observed since the introduction of immunotherapies [30–32]. Lung cancer treatment has become paradigmatic for the rapid changes in cancer therapy algorithms making prognostication increasingly difficult. Not surprisingly, a recent study has shown that the thoracic oncologists‘ tolerance of prognostic uncertainty may affect the way EOL-conversations are practiced [33]. This uncertainty amid the growing complexity of cancer treatments is also mentioned by our study’s participants, so that better information and interdisciplinary communication is strongly needed. How to address the principle of “hope for the best and prepare for the worst” in this setting of high uncertainty remains a very challenging task for GPs considering that they also often lack the information about what was said to the patient in the cancer center. This underlines the importance of strategies in dealing with uncertainty in the medical setting as proposed for education and training [34, 35]. Prior research has shown that different tools are necessary to improve the involvement of general practice in the care of cancer patients. To act as interpreters of diagnosis, treatment options, and its consequences, and engaging pro-actively in care coordination with specialists plays an important role [36]. Our study reveals that including information about EOL-topics in written communication (medical reports or the collaborative use of a digital platform) may be feasible and helpful. To improve interdisciplinary collaboration, studies implementing new strategies have shown some success: e.g. remote participation of GPs in multi-disciplinary meetings at the cancer center or integrating GPs with specific oncological skills in the center before the home return of patients [37, 38]. Still, review of the literature shows that the direct oral communication during the active phase of cancer treatment should be further developed [39]. Our results underline that in this sensitive topic not only the information flow but also the trustful GP-oncologist relationship may benefit from direct phone calls.

## Limitations

This study with a limited number of participants reflects the perspectives of general practitioners interacting with one lung cancer center in Southern Germany and consequently, the results have to be interpreted carefully. Specifics in culture and health care system have to be considered. Due to GPs’ work overload during the Covid19-pandemics the recruitment was difficult and extended over a longer period of time than planned. Possibly GPs with special interest in EOL-care and -communication chose to participate in the study.

## Conclusions

The growing complexity in modern oncology poses important challenges in GP-oncologist interaction, EOL-communication and the timely integration of palliative care. Strategies for improvement have to consider information about therapeutic advances and prognosis of patients with cancer. The written interaction in reports or digital platforms should include information about EOL-communication. Still, prognostic uncertainty may hinder the adequate provision and conduction of conversations. A trustful personal interaction through direct contact between GPs and oncologists should be encouraged as it may strengthen the interdisciplinary relationship and lead to improved coordination and goal-concordant care including the timely integration of palliative care for patients with cancer during active treatment and at the end of life.

## Electronic supplementary material

Below is the link to the electronic supplementary material.

Supplementary Material 1

Supplementary Material 2

## Data Availability

The datasets used and analyzed during this study are available from the corresponding author upon request.
