# Supplementary material for: Increasing challenges of general practitioner-oncologist interaction in end-of-life communication: a qualitative study
Source: BMC Palliat Care. 2025 Feb 20;24:48. doi: 10.1186/s12904-025-01690-w (PMC11841224; doi:10.1186/s12904-025-01690-w)
Supplement: Supplementary file 1 — Supplementary Material 1 [file 12904_2025_1690_MOESM1_ESM.docx]

**Exploring the perception of general practitioners on intersectoral communication between hospital (lung cancer center) and practice in the treatment of patients with metastatic lung cancer**

**Interview Guide**

| **Key question: Patients with metastatic lung cancer have a limited life expectancy and often alternating out- and in-patient phases. What do you discuss with these patients?**  **What topics do you discuss particularly with patients with limited life expectancy such as metastatic lung cancer?** | |
| --- | --- |
| In-depth questions:  - what topics related to treatment?  - what topics related to communication/information?  - You said XY is an issue, to what extent is palliative care/prognosis/advance car planning an issue? | Further questions:  - What experiences have you had with these discussions?  - Tell me an example of what happened with one of your patients. |
| **Key question: How do you experience the exchange of information about these patients with hospitals?** | |
| In-depth questions:  - To what extent is the information you need available?  - To what extent do you receive information about communication/conversations that took place with the patient in the hospital? | Further question:  - Can you give me an example? |
| **Key question: If you could wish for something, how would the exchange of information about patients look like?** | |
| In-depth questions:  - What information would you need?  - How or in what form would you like this information (e.g. telephone call, fax, email, discharge letter, others)? | Further questions:  - How would it be ideally?  - Tell me more. What do you mean by that? |
| **Key question: Patients are sometimes in the hospital and sometimes in your practice: What is your role/task in communicating with lung cancer patients with a limited prognosis?** | |
| In-depth questions:  - How do you describe your role in the treatment?  - Where do you see your special tasks in communication?  - Where do you see the limits of your tasks/communication?  - If role is unclear: To what extent is role clarification helpful for communicating about advance care planning, limiting therapy and resuscitation measures? | Further questions:  - What do you mean?  - Can you explain this to me? |
| **Key question: Metastatic lung cancer is not curable. As part of the treatment, the topics of prognosis, therapy limitation, resuscitation measures, advance care planning and end-of-life care come up again and again. To what extent do you talk to patients about these issues?** | |
| In-depth questions:  - How exactly do you discuss these topics? Can you give me an example?  - What is particularly important to you?  - Who should address these issues with the patient? How often? When is a good time?  - To what extent do you discuss psychological, social and spiritual issues? Who should discuss these issues? | - What exactly do you mean by that?  - I'm still thinking about what you just said. |
| **Key question: If these discussions (about prognosis, end-of-life, advance care planning, etc.) took place in the hospital, how would you like to be informed about it? How do you inform about this?** | |
| In-depth questions:  - To what extent do you want to be informed?  - In what form do you want this information? | Further question:  - What exactly does that mean in your everyday practice? |
| **Key question: Is there anything else you would like to tell me?** | |
| - Unexpected topics |  |
| **Demographic data:** form to fill out before the interview  - Gender  - Age  - Experience  - Specialization  - Communication training/palliative care training | |
