# Supplementary material for: Increasing challenges of general practitioner-oncologist interaction in end-of-life communication: a qualitative study
Source: BMC Palliat Care. 2025 Feb 20;24:48. doi: 10.1186/s12904-025-01690-w (PMC11841224; doi:10.1186/s12904-025-01690-w)
Supplement: Supplementary file 2 — Supplementary Material 2 [file 12904_2025_1690_MOESM2_ESM.docx]

**COREQ (COnsolidated criteria for REporting Qualitative research) Checklist**

| **Topic** | **Guide Questions/Description** | **Report** |
| --- | --- | --- |
| \| **Domain 1: Research team and reflexivity** \| \| --- \| | | |
| *Personal characteristics* | | |
| 1. Interviewer/facilitator   \|  \| \| --- \| | Which author/s conducted the interview or focus group?   \|  \| \| --- \| | AS and AK conducted the interviews  p.5 (methods section) |
| 2. Credentials   \|  \| \| --- \| | What were the researcher’s credentials? E.g. PhD, MD   \|  \| \| --- \| | AS: M.A.  AK: Master-degree student  MV: MD  LU: M.A. |
| 3. Occupation   \|  \| \| --- \| | What was their occupation at the time of the study?   \|  \| \| --- \| | AS: Research fellow  AK: Student  MV: Senior physician  LU: Research fellow |
| 4. Gender   \|  \| \| --- \| | Was the researcher male or female?   \|  \| \| --- \| | AS: female  AK: female  MV: male  LU: female |
| 5. Experience and training   \|  \| \| --- \| | What experience or training did the researcher have?   \|  \| \| --- \| | AK: first qualitative study  AS, MV and LU: several years in qualitative research |
| *Relationship with participants* | | |
| 6. Relationship established   \|  \| \| --- \| | Was a relationship established prior to study commencement? | Interviewers were unknown to the participants  p. 5 (methods section) |
| 7. Participant knowledge of the interviewer   \|  \| \| --- \| | What did the participants know about the researcher? e.g. personal goals, reasons for doing the research | No knowledge about interviewers  p. 5 (methods section) |
| 8. Interviewer characteristics   \|  \| \| --- \| | What characteristics were reported about the inter viewer/facilitator? e.g. Bias, assumptions, reasons and interests in the research topic | AS: Master in Nursing Science  AK: Master-degree student (Health Services Research) |
| **Domain 2: Study design** | | |
| *Theoretical framework* | | |
| 9. Methodological orientation and Theory   \|  \| \| --- \| | What methodological orientation was stated to underpin the study? e.g. grounded theory, discourse analysis, ethnography, phenomenology, content analysis | Data analysis was performed following thematic content analysis by Braun and Clarke  p. 5 (methods section) |
| *Participant selection* | | |
| 10. Sampling   \|  \| \| --- \| | How were participants selected? e.g. purposive, convenience, consecutive, snowball | Consecutive sampling |
| 11. Method of approach   \|  \| \| --- \| | How were participants approached? e.g. face-to-face, telephone, mail, email   \|  \| \| --- \| | Participants were contacted by mail and telephone; interviews were conducted by phone due to Covid-restrictions  p. 5 (methods section) |
| 12. Sample size   \|  \| \| --- \| | How many participants were in the study? | 10 participants  p. 6 (results section) |
| 13. Non-participation   \|  \| \| --- \| | How many people refused to participate or dropped out? Reasons?   \|  \| \| --- \| | ca. 100 invitations for participation via mail and phone sent out; reasons for refusal not assessed; no drop-outs |
| *Setting* | | |
| 14. Setting of data collection   \|  \| \| --- \| | Where was the data collected? e.g. home, clinic, workplace | clinic  p. 5 (methods section) |
| 15. Presence of non-participants   \|  \| \| --- \| | Was anyone else present besides the participants and researchers? | N/A |
| 16. Description of sample   \|  \| \| --- \| | What are the important characteristics of the sample? e.g. demographic data, date | Table 1 |
| *Data collection* | | |
| 17. Interview guide   \|  \| \| --- \| | Were questions, prompts, guides provided by the authors? Was it pilot tested?   \|  \| \| --- \| | A semi-structured interview guide was developed (additional file 1) and pre-tested with one physician.  p. 5 (methods section) |
| 18. Repeat interviews   \|  \| \| --- \| | Were repeat interviews carried out? If yes, how many? | No |
| 19. Audio/visual recording   \|  \| \| --- \| | Did the research use audio or visual recording to collect the data? | Audio recording  p. 5 (methods section) |
| 20. Field notes   \|  \| \| --- \| | Were field notes made during and/or after the interview or focus group? | N/A |
| 21. Duration   \|  \| \| --- \| | What was the duration of the interviews or focus group?   \|  \| \| --- \| | 25-43 minutes (average 32 minutes)  p. 5 (methods section) |
| 22. Data saturation   \|  \| \| --- \| | Was data saturation discussed?   \|  \| \| --- \| | recurring themes indicated saturation  p. 5 (methods section) |
| 23. Transcripts returned   \|  \| \| --- \| | Were transcripts returned to participants for comment and/or correction? | N/A |
| *Data analysis* | | |
| 24. Number of data coders | How many data coders coded the data? | AS and AK  p. 5 (methods section) |
| 25. Description of the coding tree | Did authors provide a description of the coding tree? | N/A |
| 26. Derivation of themes | Were themes identified in advance or derived from the data? | Use of inductive coding  to identify themes |
| 27. Software | What software, if applicable, was used to manage the data? | MAXQDA 12 software (version 12.3.9)  p. 5 (methods section) |
| 28. Participant checking | Did participants provide feedback on the findings? | No |
| *Reporting* | | |
| 29. Quotations presented | Were participant quotations presented to illustrate the themes/findings? Was each quotation identified? e.g. participant number | pp 6-11 (results section) |
| 30. Data and findings consistent | Was there consistency between the data presented and the findings? | pp 6-11 (results section) |
| 31. Clarity of major themes | Were major themes clearly presented in the findings? | pp 6-11 (results section) |
| 32. Clarity of minor themes | Is there a description of diverse cases or discussion of minor themes? | No diverse cases nor minor themes  pp 6-11 (results section) |

Developed from: Tong A, Sainsbury P, Craig J. Consolidated criteria for reporting qualitative research (COREQ): a 32-item checklist for interviews and focus groups. *International Journal for Quality in Health Care*. 2007. Volume 19, Number 6: pp. 349 – 357
